# Women health workers: improving eye care in Pakistan

**Published:** 2009-06

**Authors:** Niaz Ullah Khan, Asad Aslam Khan, Haroon R Awan

**Affiliations:** Country Director, Sightsavers International, Pakistan, House No 2, Street No 10, Sector F-7/3, Islamabad, Pakistan.; Chair, National Committee for the Prevention of Blindness; National Coordinator, National Programme for the Prevention and Control of Blindness, Pakistan.; Director, Strategic Programme Development, Sightsavers International.

**Figure:**
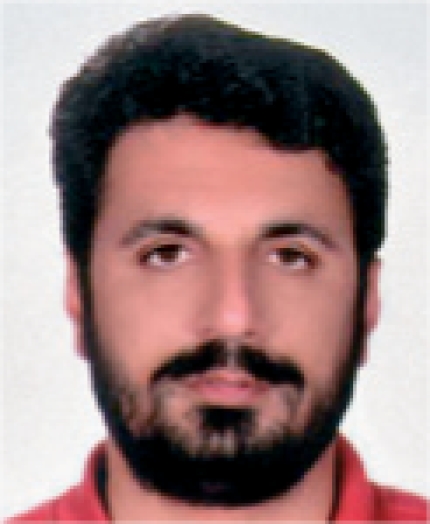


**Figure:**
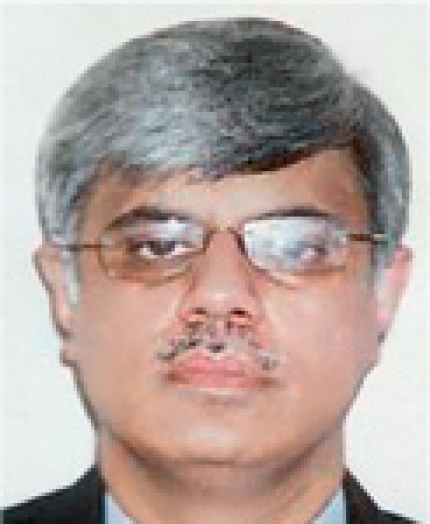


**Figure:**
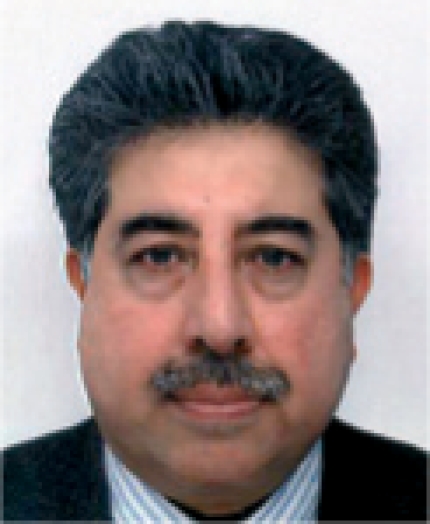


In Pakistan, female health workers (known locally as a ‘lady health workers’) have formed the backbone of the primary health care system for the past fifteen years.

These women are members of the communities they serve and are responsible for 150–200 households (around 1,000 people) each. They provide primary health care with a focus on reproductive health and family planning.

During the day, lady health workers visit women at their homes; in the evenings, community members who need help go to their local lady health worker's home (known as the ‘health house’) for health advice and basic care, including first aid.

Using women in this role is very helpful in a country such as Pakistan, where direct interaction between women and men is not encouraged. When a woman in Pakistan wants to consult a male health worker, one of her male family members is expected to accompany her. As male family members often have to work, this can make it difficult for women to make use of eye care and other health services. Lady health workers have the advantage of being able to visit women in their homes, even when male family members are at work.

## Eye care training

Although eye care has been included in lady health workers' responsibilities since the beginning, it has not been a priority. Thanks to the renewed commitment^1^ to eye care by Pakistan's national government in recent years, however, there has recently been a greater emphasis on eye care in the training of lady health workers.

Lady health workers undergo three months of classroom training in primary health care, followed by field work lasting twelve months.

**Figure:**
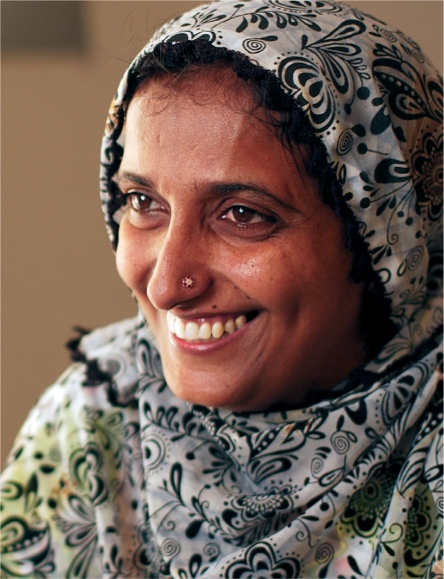
Samina became a lady health worker in 1996 and is now a master trainer. PAKISTAN

Jamshyd Masud

In the classroom, lady health workers receive between three and five days' training in primary eye care. Although the time allotted to eye care has not increased, the training has recently become more in depth and a wider range of eye conditions are covered.

During their year of field work, lady health workers interact with communities who have eye problems; they also receive one or two additional days' hands-on training in community eye care while in the field. The aim is for them to better understand common community eye health problems such as foreign body injuries, cataract, conjunctivitis, and trachoma. They also learn to perform vision screening and talk to community members about health and hygiene practices.

Until recently, training had been provided by ophthalmologists based in district community eye care programmes. In 2007, however, Sightsavers International started a national programme to develop master trainers within the National Programme of Family Planning and Primary Health Care (the programme responsible for lady health workers); these master trainers now conduct all training of lady health workers in Pakistan. A training manual in the local Urdu language has been developed in consultation with all parties and was approved by the national eye health committee.

On completion of their primary eye care training, lady health workers are able to perform basic vision assessments (they are given E charts to use); they are also able to deal with conjunctivitis and foreign body injuries. They can screen patients for cataract, trachoma, low vision, and childhood blindness and when necessary they refer community members to nearby eye care services.

## Impact

In the Federally Administered Tribal Areas (FATA), where the new eye care curriculum was piloted and where it had been taught for five consecutive years (2001–2005), we found that lady health workers dealt with more than three times as many eye patients as colleagues in other provinces who had not yet received the training. The programme will be evaluated on a national level in November 2009.

**Useful facts**

- There are 80,000 lady health workers in Pakistan at present; the government has committed to add another 20,000 by the end of 2009.
- Lady health workers are required to have at least eight years of schooling, although most have ten years. The position is advertised locally and applicants are interviewed.
- On average, a lady health worker will visit five households every day; her aim is to visit each house once a month. Workers will visit pregnant women and those with newborn babies two or three times per month.
- At present, a stipend of about US$ 40 per month is paid to each health worker; their remuneration package is currently under review.
- Supervisors manage groups of 15–20 lady health workers each. They spend a day with each health worker per month to assess her work. They also assess lady health workers' knowledge and skills against the prescribed checklist developed by the programme.
